# Earthworms accelerate rice straw decomposition and maintenance of soil organic carbon dynamics in rice agroecosystems

**DOI:** 10.7717/peerj.9870

**Published:** 2020-09-17

**Authors:** Ke Song, Lijuan Sun, Weiguang Lv, Xianqing Zheng, Yafei Sun, William Terzaghi, Qin Qin, Yong Xue

**Affiliations:** 1 Institute of Eco-Environmental and Plant Protection, Shanghai Academy of Agricultural Sciences, Shanghai, China; 2 Department of Biology, Wilkes University, Wilkes-Barre, PA, USA

**Keywords:** Earthworms, Rice residues, SOC, Aggregate-associated carbon, Soil basal respiration, Enzyme activities

## Abstract

**Background:**

To promote straw degradation, we inoculated returned farmland straw with earthworms (*Pheretima guillelmi*). Increasing the number of earthworms may generally alter soil organic carbon (SOC) dynamics and the biological activity of agricultural soils.

**Methods:**

We performed soil mesocosm experiments with and without earthworms to assess the decomposition and microbial mineralization of returned straw and soil enzyme activity across different time periods.

**Results:**

When earthworms were present in soil, the surface residues were completely consumed during the first four weeks, but when earthworms were absent, most of the residues remained on the soil surface after 18 weeks. On day 28, the SOC content was significantly higher in the treatment where both earthworms and residue had been added. The SOC content was lower in the treatment where earthworms but no residue had been added. The organic carbon content in water-stable macroaggregates showed the same trend. During the first 14 weeks, the soil basal respiration was highest in the treatments with both residues and earthworms. From weeks 14 to 18, basal respiration was highest in the treatments with residues but without earthworms. We found a significant positive correlation between soil basal respiration and soil dissolved organic carbon content. Earthworms increased the activity of protease, invertase, urease and alkaline phosphatase enzymes, but decreased β-cellobiohydrolase, β-glucosidase and xylosidase activity, as well as significantly reducing ergosterol content.

**Conclusion:**

The primary decomposition of exogenous rice residues was mainly performed by earthworms. Over a short period of time, they converted plant carbon into soil carbon and increased SOC. The earthworms played a key role in carbon conversion and stabilization. In the absence of exogenous residues, earthworm activity accelerated the decomposition of original organic carbon in the soil, reduced SOC, and promoted carbon mineralization.

## Introduction

Crop residue is one of the main wastes generated by agricultural production, with 100–200 billion tons produced globally every year. It is an important renewable resource with great comprehensive utilization value, and is rich in nitrogen, phosphorus, potassium and other trace elements. The main method of utilizing crop residue is to return it to the field. The United States produces 450 million tons of crop residue annually and returns 68% of that total to the field. The United Kingdom returns 73% of its total crop residue to the field. China, the world’s largest producer of crop residue with about 900 million tons generated annually, returns 78% of that to the field (Sun et al., 2018). Crop residue returns eliminate the air pollution created by burning residue, increase soil organic carbon (SOC) and promote microbial vigor (Benbi & Senapati, 2010; Verhulst et al., 2011; Song et al., 2019). However, due to the slow decomposition of returned residue, returning it also leads to an increase in soil pathogens, aggravation of crop diseases, reduced seedling numbers and ultimately reduces crop yield (Yadav et al., 2005; Prasad et al., 2016).

As one of the most important soil animals in terrestrial ecosystems, earthworms play a key role in removing plant residues and other organic materials from the soil surface by converting them into soil carbon (Martin, 1991). It has been shown that adding earthworms to surface residues significantly accelerates their degradation, even in those with high C/N residue (Frouz et al., 2014). Additionally, earthworms promote the transfer of plant carbon from residues to soil aggregates (Six et al., 2004; Hedde et al., 2013). However, it has been debated whether earthworms increase SOC storage or promote SOC mineralization. Several studies have shown that earthworms increased the binding of residual carbon in soil aggregates, reducing long-term decomposition (Chan, 2001; Bossuyt, Six & Hendrix, 2005; Pulleman et al., 2005b; Zhang et al., 2013). Zhang et al. (2013) proposed the concept of a “carbon trap,” where earthworms stabilize a greater proportion of plant residue carbon in soil aggregates instead of mineralizing the residue as carbon dioxide (CO_2_). In this process, most of the C flows rapidly into the earthworm gut, where it is converted into stabilized forms and stored in casts. Several other studies found that earthworms did promote the mineralization of SOC (Binet et al., 1998; Edwards, 2004; Coq et al., 2007), increasing CO_2_ emissions from soil with more long-term SOC decomposition (Lubbers et al., 2013; Wachendorf et al., 2014). After a 750-day experimental study, Lubbers, Pulleman & Van Groenigen (2017) proposed that the organic carbon content was lower because the presence of earthworms increased the decomposition rate of total organic matter (OM). They conjectured that earthworms stimulated the mineralization of carbon in newly added plant residues and old OM, not the sequestration in stable aggregates.

Based on these arguments, we wanted to explore how earthworms promote the rapid degradation of residues. We know that earthworms can accelerate decomposition through a series of activities, including crushing, feeding, digesting and burrowing, that help convert fresh residues to soil humus (Marone et al., 2017). But these transforming activities can only transport, break, or directly convert plant carbon into soil carbon. It is uncertain whether the activities of microorganisms and enzymes in the earthworm gut can stimulate the quantity and activity of exogenous microorganisms in the surrounding soil. Parle (1963) first reported the presence of microorganisms in the gut of earthworms, and subsequent studies used direct culture methods to investigate gut microbiota (Karsten & Drake, 1995; Garg, Kaushik & Dilbaghi, 2006). Various gut microbes that produce digestive enzymes (such as amylases, proteases, lipases and cellulases) have been found to enhance the biodegradation of OM in earthworm guts (Aira, Monroy & Domínguez, 2006). Other studies suggested that earthworms’ extensive feeding and burrowing activities (Curry & Schmidt, 2007) altered the quantities and activities of microorganisms in soil (Brown, Barois & Lavelle, 2000; Ernst et al., 2008; Azadeh & Zarabi, 2014) and promoted the reproduction and activity of soil microorganisms (Sinha et al., 2011; Bhat & Limaye, 2012). Several studies have suggested that microbes provide food for earthworms (Dash, Mishra & Behera, 1979) and that earthworms feed, disperse and kill soil microbes (Zhang & Xu, 1990), reducing their numbers.

From previous studies, we hypothesized that earthworms accelerate the degradation of residues mainly through a series of biological activities, store the carbon from plant residues in soil aggregates in the form of casts, and reduce the ability of soil microbes to decompose mineralized soil carbon by feeding, dispersing and killing them, increasing soil carbon sequestration. In order to test these hypotheses, we added rice residues to the surface of soil with and without earthworms. We confirmed the ability of the earthworms to promote the rapid decomposition of residues and compared the quantities of soil aggregates and SOC sequestration. We also measured changes in soil chemical properties and enzyme activity.

## Materials and Methods

### Collecting materials

We performed our laboratory experiment at the Shanghai Academy of Agricultural Sciences. Test soil was collected from the Sanxing Experimental Station (SES) on Chongming Island (121°33′47″E, 31°41′20″N), China. The station had implemented a rice (*Oryza sativa* L.)–wheat (*Triticum aestivum* L.) rotation system at this site for nearly 10 years. We collected test soil from the 0–20 cm depth layer of a rice plot (the area is 15 × 32 = 480 m^2^) after harvest on 26 November 2017. The collected soil was Typic Fluvaquents and had 15.21 g·kg^−1^ of OM, 0.94 g·kg^−1^ of total nitrogen, 81.62 mg·kg^−1^ of alkali-hydrolyzable nitrogen, 53.53 mg·kg^−1^ of available phosphorus, 109.35 mg·kg^−1^ of available potassium, and a pH of 8.30 (a 5:1 water-to-soil ratio). We transported the collected soil back to the laboratory after removing debris, such as stones and animal and plant residues. The samples were air-dried, ground, passed through a 2 mm sieve, and stored for later use.

In this study, we used rice straw for the test residue. Rice residues were collected from the same rice field where the soil had been collected following harvesting. They were air-dried for two weeks under natural ventilation conditions, then broken into approximately 1 cm pieces with a micro-mill and stored for later use. The rice residue had a C/N of 53.11, and was 36.43% cellulose, 22.52% hemicellulose and 18.69% lignin.

We used the earthworm species *Pheretima guillelmi* (Zhang et al., 2001) which is an endogeic ecotype. The earthworms were collected from rice fields during harvest and were cultivated in the laboratory in polyethylene barrels containing soil (collected from the same rice plot where the earthworms had been collected) and rotting rice residues. We selected individuals weighing 2.5–3.5 g prior to the experiment, and expurgated their guts for 48 h using the filter paper method to remove excrement that could affect the test results.

### Experimental design

We conducted a mesocosm experiment over 126 days (Tiunov & Scheu, 2004; Frouz et al., 2014). The four applied treatments were combinations with and without earthworms and rice residues covering the soil surface: −Rr−Ew (no surface rice residues and no earthworms), −Rr+Ew (no surface rice residues but with earthworms), +Rr−Ew (with surface rice residues but no earthworms), and +Rr+Ew (with surface rice residues and with earthworms). Each treatment was replicated three times. Each mesocosm was placed in a 4-l circular polyethylene container with soil from the rice plot. The polyethylene containers each had a diameter of 25 cm and a height of 10 cm. We placed 2,000 g of air-dried soil in each container and 20 g of rice residues evenly on the surfaces of the treatment soils. After the soil and residues were prepared, we moistened the soil by spraying 800 ml of distilled water into each container. We weighed the containers every three days and watered them every two weeks to keep a constant moisture level. Five selected earthworms were placed in each container for the treatments with earthworms. The density of inoculated earthworms in this study was higher than the average for surface soil, at about 0–1 earthworm per 3 kg soil in the 0–20 cm soil layer. However, this higher inoculation density could offset the impact of short-term culture experiments to some extent by accelerating their effect on soil and residues (Hale et al., 2008). We covered each polyethylene container with a polyethylene lid with ten holes to allow air exchange, reduce water evaporation, and prevent the earthworms from escaping. The room temperature was maintained at 25 °C. The mesocosms were examined weekly to confirm whether the earthworms were alive and active. Dead earthworms were removed and replaced with another of the same size.

### Soil analyses

The experiment began on 28 April 2018 and ended on 30 August 2018. During the experiment, we measured soil respiration with an infrared gas analyzer every week (Heinemeyer et al., 1989) and collected 20 g of soil to determine dissolved organic carbon (DOC). We only collected from the top 0–5 cm of soil and after removing rice residues covering the soil surface. After removing rice residues from the surface, we collected 200 g soil samples from the top 0–5 cm on 25 May 2018 (Day 28) and 30 August 2018 (Day 126). The rice residue was replaced after collecting the soil samples. The soil sample was divided into two parts, and one was directly used to determine ergosterol content and the activity of protease, invertase, urease, alkaline phosphatase, β-cellobiohydrolase, β-glucosidase, xylosidase and chitinase enzymes. We extracted and tested the ergosterol using the method described by Šnajdr et al. (2008), a Waters Alliance high performance liquid chromatography system (Waters, Milford, MA, USA), and methanol as the mobile phase at a flow rate of 1 ml/min and UV detection at 282 nm. We measured the protease, invertase, urease and alkaline phosphatase activity using the method described by Zhou (1987). We used ninhydrin colorimetry to confirm protease activity, the 3,5-dinitrosalicylic acid colorimetric method to confirm invertase activity, the indophenol blue colorimetric method to confirm urease activity, and phenyl phosphate sodium colorimetry to confirm alkaline phosphatase activity. We determined β-cellobiohydrolase, β-glucosidase, xylosidase and chitinase activity using Marx, Wood & Jarvis’s (2001) fluorescent microplate enzyme assay method. We used methylumbelliferone (MUB)-β-_D_-cellobioside as a substrate for β-cellobiohydrolase, MUB-β-_D_-glucopyranoside for β-glucosaccharase, MUB-β-_D_-xylopyranoside for xylosidase, and MUB-N-acetyl-β-_D_-glucosaminide for chitinase. The other part of the soil sample was naturally air-dried, and the soil aggregate composition was determined using wet sieving. The different particle sizes obtained by a DIK-2001 soil aggregate analyzer (RKC Instrument Inc., Saitama, Japan) were macroaggregates (>0.25 mm), microaggregates (0.25–0.053 mm), and silt and clay (<0.053 mm) (Song et al., 2017). We then separately ground and passed the remaining air-dried soil and soil aggregates through a 0.149-mm sieve to measure SOC (Lu, 1999).

### Statistical analyses

After using Microsoft Excel 2010 to collate the data, we used the SPSS 17.0 software package for variance analysis. We performed a significant difference test using one-way ANOVA at a *P* < 0.05 level and a two-way ANOVA with repeated measures. We used the Tukey method to make multiple comparisons across the different treatments, and Origin 8.0 to process the graphics.

## Results

### Soil organic carbon

**Figure 1:**
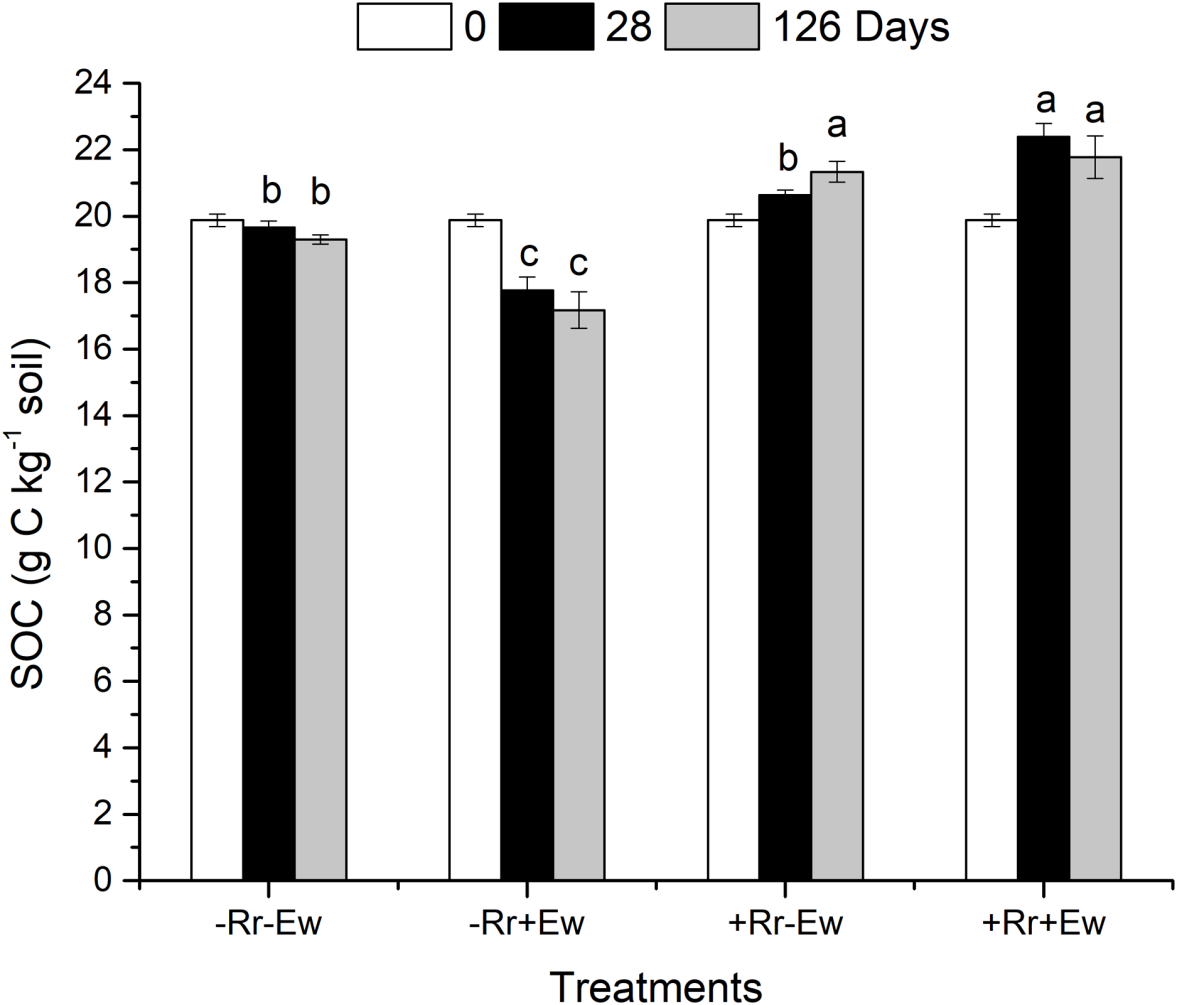
Soil organic carbon (SOC) in different treatments of residues and earthworms at two different time-points. The treatments were: −Rr−Ew (without surface rice residues or earthworms), −Rr+Ew (without surface rice residues with earthworms), +Rr−Ew (with surface rice residues without earthworms), and +Rr+Ew (with surface rice residues and with earthworms). Values are means ± SD, *n* = 3. Treatments indicated by the same letter were not significantly different at *P* ≤ 0.05 based on one-way ANOVA.

On the 28th day of the experiment, the surface residues of the +Rr+Ew treated soil (with both earthworms and residues) were completely degraded (no residues were visible to the naked eye). However, the residues still covered the surface of the +Rr−Ew treated soil (with residues and no earthworms). At the same time, the surface layers of the −Rr+Ew samples (no residues and with earthworms) were covered with soil aggregates, while no soil aggregates were observed in the −Rr−Ew samples (no residues and no earthworms). On the 126th day, much residues still covered the surface of the +Rr−Ew treated soil with a large number of fungal hyphae attached to the residues. As shown in Fig. 1, compared to the 0th, the SOC content increased in the treatments with residues, and decreased in those without residues on both the 28th and 126th days. On the 28th day, the SOC content in the −Rr+Ew samples was significantly lower than in the −Rr−Ew samples. However, the +Rr+Ew samples had significantly higher SOC than the +Rr−Ew samples. On the 126th day, the SOC content was much lower in the −Rr+Ew samples than in the −Rr−Ew samples. On the 28th day, the SOC content continued to increase in the +Rr−Ew samples, but decreased in the +Rr+Ew samples, and there was no significant difference between treatments with and without earthworms.

### Water-stable aggregates

**Figure 2:**
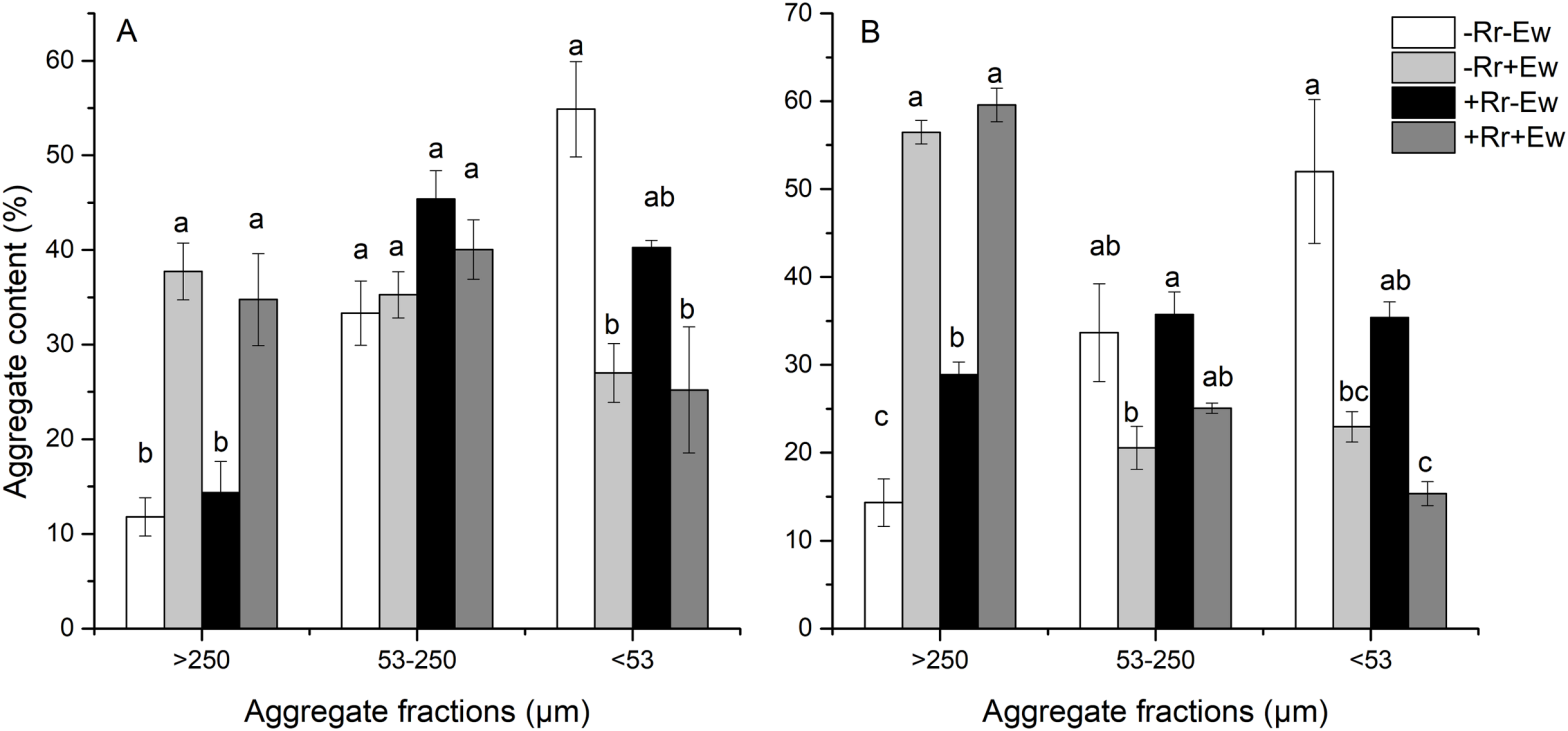
Aggregate content on (A) Day 28 and (B) Day 126 of residue and earthworm treatments. Treatments are described in Fig. 1. Values are means ± SD, *n* = 3. Treatments indicated by the same letter within the same graph were not significantly different at *P* ≤ 0.05 based on one-way ANOVA.

We found that earthworm activity strongly influenced the composition of water-stable aggregates. As shown in Fig. 2, the content of water-stable macroaggregates (>250 μm) on the 28th and 126th days was significantly higher in treatments with earthworms than in those without earthworms. On the 28th day (Fig. 2A), the added residues did not significantly affect the macroaggregates or microaggregates between treatments. However, the −Rr+Ew samples had the highest amount of macroaggregates, the +Rr−Ew samples had the highest amount of microaggregates, and top soil in the treatments without earthworms had more silt and clay than those with earthworms. On the 126th day (Fig. 2B), the +Rr−Ew samples had a higher amount of macroaggregates than the −Rr−Ew samples, but we found no significant difference between the −Rr+Ew and +Rr+Ew treatments. There were significantly fewer microaggregates, silt, and clay in the treatments with earthworms than those without earthworms.

### Aggregate-associated carbon

**Figure 3:**
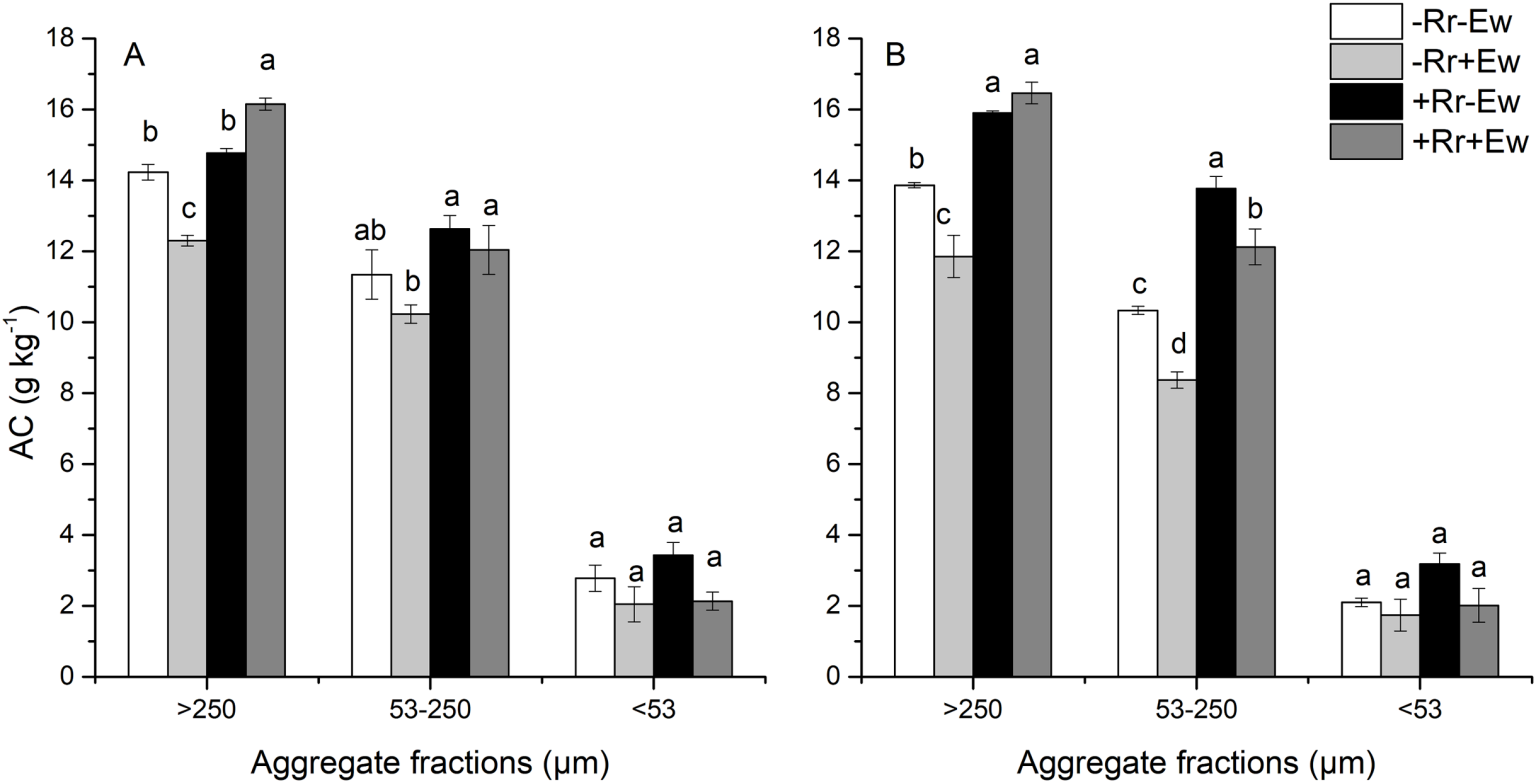
Aggregate-associated carbon (AC) on (A) Day 28 and (B) Day 126 of residue and earthworm treatments. Treatments are described in Fig. 1. Values are means ± SD, *n* = 3. Treatments indicated by the same letter within the same graph were not significantly different at *P* ≤ 0.05 based on one-way ANOVA.

As shown in Fig. 3, macroaggregates had the highest SOC, followed by microaggregates and then silt and clay. The presence of residues increased the amount of carbon found in macroaggregates and microaggregates. The effect of earthworm activity on aggregate-associated carbon (AC) was two-sided. On the 28th day, the amount of SOC associated with macroaggregates was significantly higher (9.35%) in the +Rr+Ew treatments than in the +Rr−Ew treatments. In the treatments without residues, macroaggregate-associated carbon was significantly lower (15.72%) in the −Rr+Ew treatments than in the −Rr−Ew treatments. Conversely on the 126th day, macroaggregate-associated carbon was slightly higher in the +Rr+Ew samples than in the +Rr−Ew samples, although this difference was not significant. However, macroaggregate-associated carbon was significantly lower (16.95%) in the −Rr+Ew samples than in the −Rr–Ew samples. There was no significant difference in microaggregate-associated carbon between the two treatments with residues, but it was significantly lower in the −Rr+Ew group than in the other treatments on the 28th day. On the 126th day, microaggregate-associated carbon was significantly lower in the treatments with earthworms than in those without. We found no significant variations in the SOC content of silt and clay across treatments at the two sampling time points.

### Basal respiration and DOC

**Figure 4:**
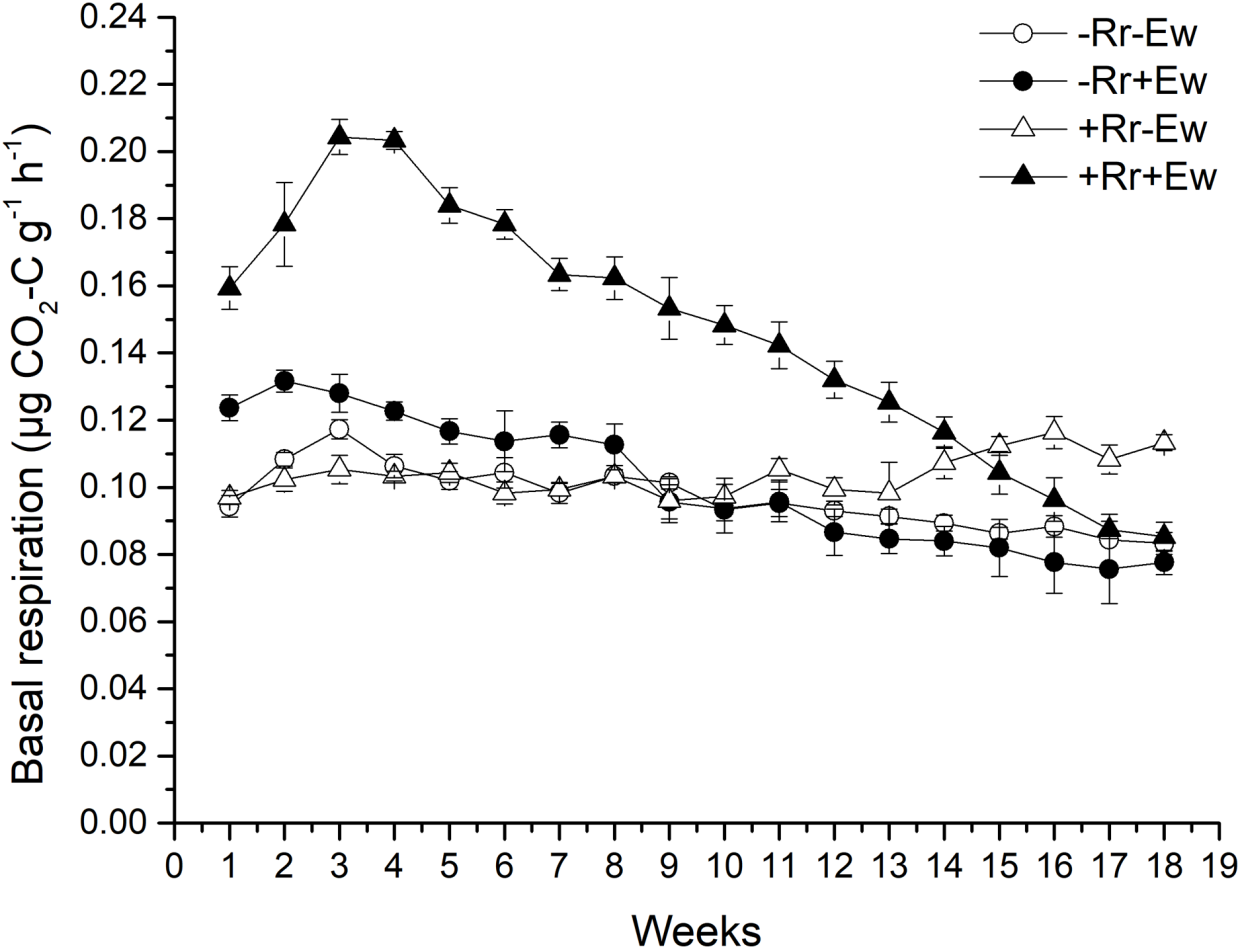
Soil basal respiration after residue and earthworm treatments. Values are means ± SD, *n* = 3, measured weekly. Treatments are described in Fig. 1.

**Figure 5:**
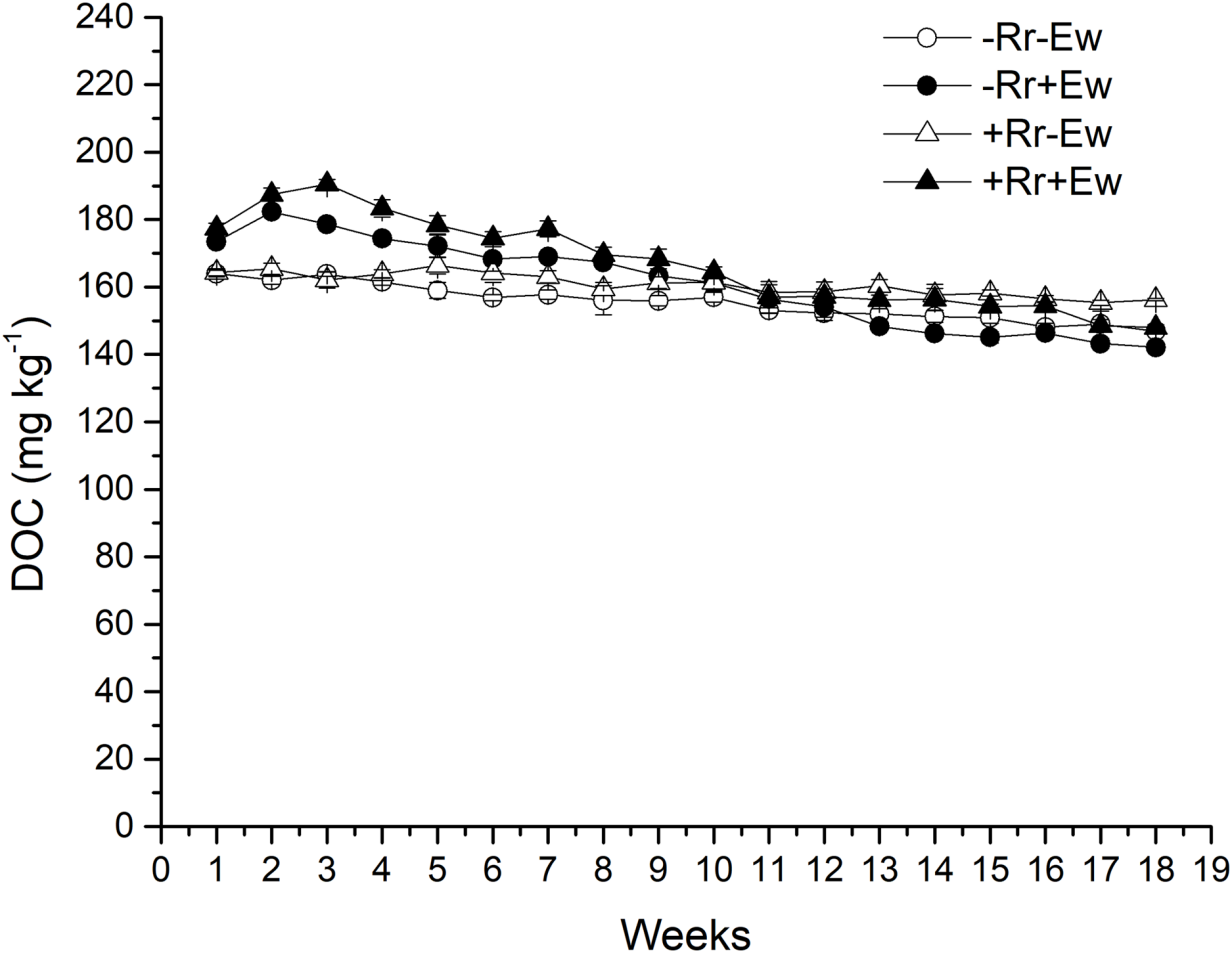
Dissolved organic carbon (DOC) after residue and earthworm treatments. Values are means ± SD, *n* = 3, measured weekly. Treatments are described in Fig. 1.

Earthworm activity and the presence of residues had a significant impact (*P* < 0.05) on soil basal respiration. During the first 14 weeks, basal respiration was highest in the +Rr+Ew samples (Fig. 4). Following that, basal respiration was highest in the +Rr−Ew samples. In the absence of residues, basal respiration was initially higher in the −Rr+Ew samples than in the −Rr−Ew samples, but decreased in the −Rr+Ew samples after 10 weeks. Without earthworms, the presence of residues did not initially increase soil basal respiration, but +Rr−Ew samples had much higher respiration than −Rr−Ew samples after 10 weeks. The presence of earthworms also strongly influenced soil DOC (Fig. 5), which was significantly higher during the first 10 weeks in treatments with earthworms than that in those without earthworms. +Rr−Ew samples had the highest DOC, followed by +Rr+Ew, −Rr−Ew and then −Rr+Ew.

### Enzyme activities

Enzyme analyses revealed that earthworm activity and presence of residues had a strong impact on soil enzymatic activity (Table 1). The presence of residues had a positive impact on the activity of chitinase and all other enzymes. In particular, invertase, β-cellobiohydrolase, β-glucosidase, and xylosidase activity increased significantly, and were higher on day 126 than on day 28. Earthworms significantly increased protease, invertase, urease, and alkaline phosphatase activity, but had a negative impact on β-cellobiohydrolase, β-glucosidase and xylosidase activity. The −Rr+Ew treatment had the lowest enzymatic activity. Additionally, the enzymatic activity was much lower on day 126 than on day 28 in the treatments with earthworms. The interactions between residues and earthworms increased protease, invertase, urease, and alkaline phosphatase activity, but did not have a significant effect on chitinase activities.

**Table 1:** Protease, invertase, urease, alkaline phosphatase, β-cellobiohydrolase, β-glucosidase, xylosidase, and chitinase enzyme activity (nmol g^−1^ h^−1^) in soil at 28 and 126 days of residue and earthworm treatment.

Enzyme	Days	−Rr−Ew	−Rr+Ew	+Rr−Ew	+Rr+Ew
Protease	28	74.45 ± 3.73d	113.06 ± 2.65b	90.01 ± 3.35c	140.57 ± 8.14a
	126	62.84 ± 6.67c	88.96 ± 6.61b	92.82 ± 4.88b	112.73 ± 3.32a
Invertase	28	968.45 ± 21.62d	1400.89 ± 123.17c	1811 ± 27.14b	2342.28 ± 56.23a
	126	764.31 ± 8.14d	1663.44 ± 49.19c	2087.10 ± 49.96b	2532.77 ± 113.67a
Urease	28	42.83 ± 3.20d	88.93 ± 3.64b	60.79 ± 3.42c	102.69 ± 3.90a
	126	43.55 ± 4.43d	90.43 ± 4.57b	71.99 ± 3.87c	104.43 ± 4.03a
Alkaline phosphatase	28	78.67 ± 3.41d	163.36 ± 5.44b	130.05 ± 9.00c	188.65 ± 8.53a
	126	80.37 ± 3.64c	156.89 ± 4.29b	152.86 ± 2.86b	192.72 ± 9.01a
β-cellobiohydrolase	28	86.49 ± 7.64c	52.32 ± 5.30d	125.46 ± 3.05a	108.65 ± 2.74b
	126	74.28 ± 5.10b	44.93 ± 2.34c	147.75 ± 7.37a	63.31 ± 4.67b
β-glucosidase	28	145.68 ± 4.35c	88.12 ± 4.80d	211.32 ± 9.66a	172 ± 12.13b
	126	122.37 ± 5.18b	84.03 ± 1.40c	257.51 ± 15.94a	133.72 ± 4.93b
Xylosidase	28	72.11 ± 2.87b	43.62 ± 7.03c	104.6 ± 6.02a	85.58 ± 1.80b
	126	58.61 ± 4.93b	35.46 ± 4.07c	155.02 ± 6.31a	63.62 ± 4.93b
Chitinase	28	123.71 ± 3.47a	121.36 ± 2.82a	130.51 ± 5.18a	119.22 ± 5.19a
	126	117.39 ± 6.18a	119.6 ± 10.04a	126.05 ± 7.19a	124.36 ± 5.41a

**Note:**

Treatments are described in Fig. 1. Values are means ± SD, *n* = 3. Treatments indicated by the same letter were not significantly different at *P* ≤ 0.05 based on one-way ANOVA.

## Discussion

### SOC, aggregates and AC

Our results showed that the presence of residues increased the SOC content, and that earthworm activity significantly accelerated the degradation of rice residues. The residues were completely degraded within the first four weeks in the treatments with earthworms, while residues still covered the soil surface in the treatment with residues and without earthworms. Previous studies showed that SOC is mainly derived from plant carbon and that earthworms promote the conversion of plant residues to soil carbon through crushing, feeding, digestion and burrowing (Binet et al., 1998). We found that the SOC content was highest in the +Rr+Ew treatment on day 28. Because it was higher than in the +Rr−Ew treatment, this proved that earthworms had promoted the conversion of plant carbon to soil carbon and accelerated residue degradation. However, we found no significant difference in SOC content between the two treatments with and without earthworms on day 128, showing that residue carbon was also converted to soil carbon over time even without earthworms (Castellanos-Navarrete et al., 2012). When no residues were present, the SOC content with earthworm treatment was significantly lower than treatment without earthworms on both days 28 and 126, indicating that earthworm activity reduced SOC and increased carbon mineralization (Baker et al., 2007). A field study showed that a soil’s total carbon content was lower after five months of earthworm inoculation than without earthworm inoculation (49.3 g·c·kg^−1^ vs. 50.3 g·c·kg^−1^, *p* = 0.004) (Coq et al., 2007). We also analyzed the aggregate and AC content across our different treatments. The results showed that earthworm activity significantly increased soil macroaggregates, which was in agreement with those of previous studies (Pulleman et al., 2005a; Jastrow, Amonette & Bailey, 2007; Schmidt et al., 2011). Moreover, our research also determined that the content of water-stable macroaggregates in the −Rr+Ew treatments was higher than that of the +Rr+Ew treatments on day 28. This may be because −Rr+Ew earthworms digested more soil and discharged more casts due to the lack of food. In contrast, the content of soil macroaggregates was higher in samples with residues than in those without residues by day 126, perhaps due to the significant decrease in earthworm activity from the lack of food (Hedde et al., 2013). The content of microaggregates in samples with earthworms was lower than in those without earthworms. This was mainly due to the higher density of earthworms in the simulation experiment creating more macroaggregates in a short period of time by discharging a large amount of earthworm casts (Crittenden et al., 2014). In addition to the effects on water-stable aggregates, we found that earthworm activity strongly influenced AC. On day 28 of the +Rr−Ew and +Rr+Ew treatments, the content of macroaggregate-associated carbon was higher in the +Rr+Ew samples than in the +Rr−Ew samples, indicating that the earthworms converted residue carbon into AC by excreting casts. In the absence of residues, the level of macroaggregate-associated carbon was lower in treatments with earthworms than in those without earthworms. This indicated that earthworm activity decreased SOC content without the input of exogenous plant carbon, which increases carbon mineralization (Chevallier et al., 2001). Although the aggregates formed by earthworm casts could physically maintain the levels of organic carbon, in the absence of exogenous plant carbon input, the main function of earthworm activity is to stimulate the mineralization of soil carbon (Marhan et al., 2007). Moreover, the AC content in microaggregates and silt and clay decreased, indicating that earthworm activity had accelerated the mineralization of SOC in these aggregate fractions. Zhang et al. (2013) suggested that earthworms activate a large proportion of carbon to maintain their metabolism, indicating that in soil with lower carbon levels, carbon mineralization is the main process and carbon stabilization occurs less frequently. However, in soil with higher carbon content, the carbon used during earthworm metabolism is a small part of the mineralizable carbon pool and the stimulation of CO_2_ emission was relatively lower, suggesting that carbon stabilization may be the main focus. Our results revealed that earthworms could convert plant carbon into soil carbon when they were fed residues that covered the soil surface, and excreted casts to form soil aggregates that physically maintained SOC levels. During this process, earthworms played a dual role. They accelerated the degradation of rice residues and converted them into soil carbon (in the absence of earthworms, microorganisms can also decompose residues and convert residue carbon to soil carbon, but this process is longer). Additionally, earthworm casts formed soil aggregates that provide physical protection for their own converted carbon, making the organic carbon content in the macroaggregates higher than in the soil without earthworms. In the absence of exogenous plant carbon input, earthworms provided nutrients for microorganisms by self-decomposing and excreting carbohydrates, accelerating the degradation and consumption of original organic carbon in the soil (Edwards, 2004), although the aggregates formed by earthworm casts could also maintain organic carbon levels (Knowles, Ross & Gorres, 2016). However, the SOC content in macroaggregates was still lower than in soil without earthworms, indicating that in the absence of exogenous carbon input, earthworms activated the original carbon in the soil and increased the mineralization rate (Lubbers et al., 2013).

### Soil basal respiration and DOC

**Figure 6:**
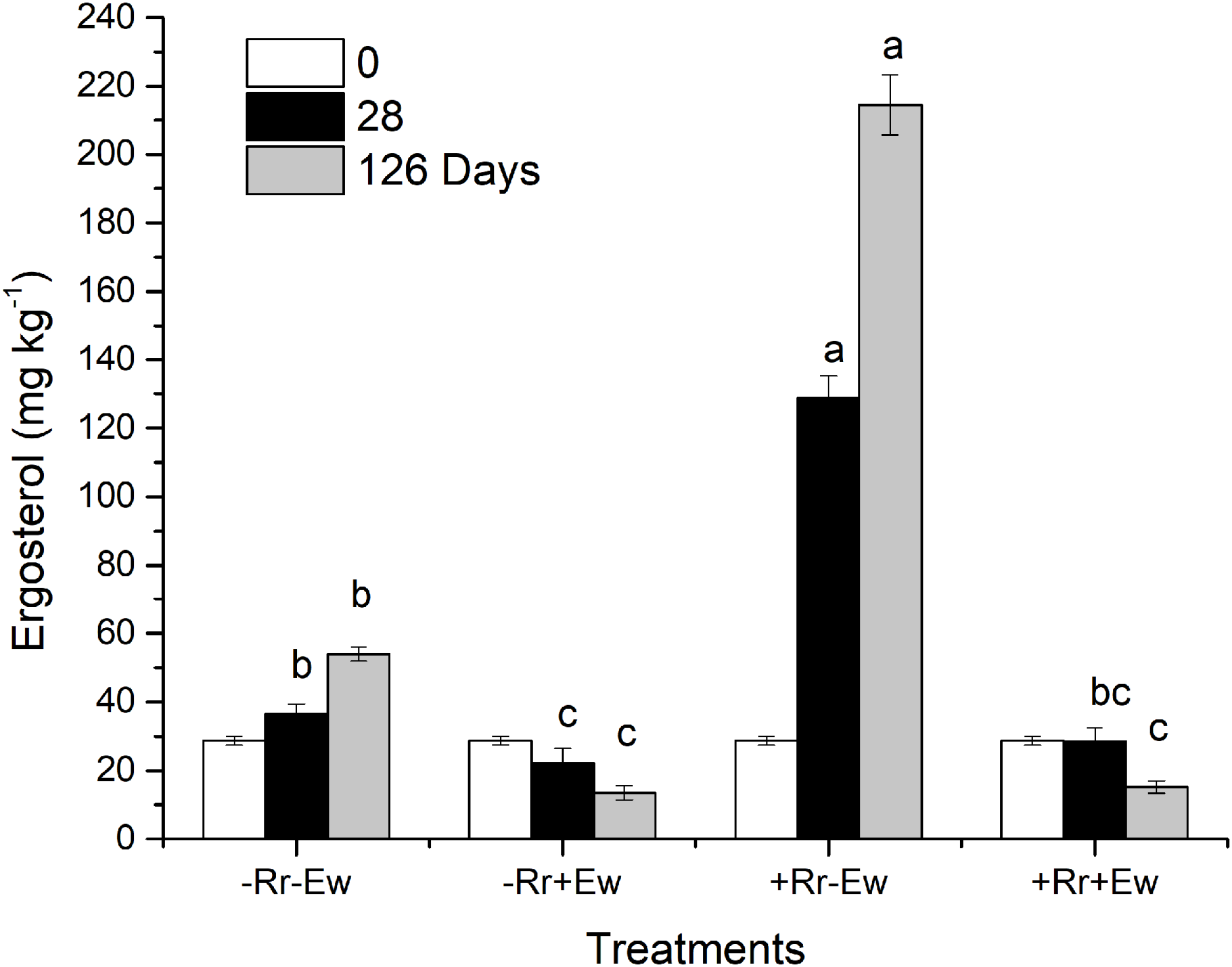
Ergosterol content in different treatments of residues and earthworms at two different time-points. Treatments are described in Fig. 1. Values are means + SD, *n* = 3. Treatments indicated by the same letter were not significantly different at *P* ≤ 0.05 based on one-way ANOVA.

So how do earthworms accelerate the conversion of residue carbon into soil carbon? Other studies have suggested that plant carbon is converted into soil carbon mainly by microorganisms and digestive enzymes in the guts of earthworms feeding on plant residues (Hong, Lee & Chung, 2011; Kim et al., 2011; Blouin et al., 2013). However, different studies found that earthworms accelerate the degradation of plant residues mainly by stimulating microorganisms in the soil (Munnoli, Da Silva & Saroj, 2010; Aira & Dominguez, 2011; Yadav & Garg, 2011). Our results showed that in the presence of earthworms, the soil basal respiration rate and chemical oxygen demand (COD) significantly increased. There was a significant positive correlation between the basal respiration rate and COD in the same treatment (−Rr+Ew: *Y* = 668.62*X* + 93.14, *R* = 0.979; +Rr+Ew: *Y* = 525.76*X* + 96.31, *R* = 0.864), proving that earthworm activity accelerated carbon mineralization and increased CO_2_ emissions. The increase of COD in earthworm casts may have increased soil basal respiration. Yadav & Garg (2011) found that earthworms gradually altered the biological, physical and chemical state of residue, lowering the C:N ratio and increasing the surface area exposed to microorganisms by feeding, comminuting, and digesting OM. Lavelle (1988) suggested that an increase in soil respiration was associated with higher concentrations of water-soluble carbohydrates in earthworm digestive tract and casts. However, during later stages, soil basic respiration and COD were shown to decrease, indicating a reduction in soil mineralized organic carbon (Amador, Görres & Savin, 2003). This was mainly due to a lack of food for earthworms since no exogenous residues were provided. The results of a 126-week experiment from Frouz & Šimek (2009) also showed an increase in soil respiration soon after earthworms entered the soil, although soil respiration was ultimately reduced because earthworm casts provided nutrients and stimulation for the microbes only in the early stages. The growth and reproduction of microorganisms accelerated organic carbon decomposition, but respiration intensity decreased with the consumption of unstable OM and the accumulation of microbial by-products. This indicated earthworms accomplished the primary decomposition of rice residues, converting refractory residues into carbohydrates that could be easily used by microorganisms, provide nutrients, and accelerate the residue degradation process. We also measured the ergosterol levels across the different treatments. Our results (Fig. 6) showed that the treatments with earthworms had significantly lower ergosterol content than those without earthworms, indicating that the proportion of fungi to bacteria significantly decreased in the treatments with earthworms, mainly due to the earthworms’ consumption of humus, animal waste, soil fungi and bacteria (Aghababaei, Raiesi & Hosseinpur, 2014). During the experiment, we observed with the naked eye that there were many fungal hyphae and weeds on the surface of soil without earthworms, but only earthworm casts on the soil surface with earthworms. This suggested that the fungus was inhibited in the presence of earthworms. On days 28 and 126, ergosterol was significantly higher in the treatment with residues and without earthworms than in any other treatment, which suggests that this treatment had the most fungus. This may have been due to the rice residues providing nutrition for the fungus, which then promoted the decomposition of rice residues. This also indicated that microorganisms (particularly fungi, which were previously considered to be the main microorganism decomposing rice residues) were not the main driving force for the degradation of residues when earthworms were present. We attributed this to the conversion of rice residues into unstable carbon sources during earthworm feeding and digestion providing nutrients for microbes, especially for the bacteria that accelerate the mineralization of unstable carbon (Aira, Pérez-Losada & Domínguez, 2019). However, in the absence of earthworms, fungi mainly carried out the degradation of rice residues at a slower rate (Song et al., 2019). Wardle (2002) also found that earthworm activity transformed soil ecosystems with slower fungal-based nutrient turnover into systems dominated by bacteria and rapid nutrient turnover. Further evidence supporting this view was obtained by measuring the enzyme activities in the soil.

### Enzyme activities

Our soil enzyme activity measurements showed that earthworms and residues had different effects on enzymatic activities. The presence of residues increased the activities of almost all enzymes present in the soil, especially those of invertase, β-cellobiohydrolase, β-glucosidase and xylosidase, but with the sole exception of chitinase. This suggested that the presence of residues significantly increased the activity of enzymes involved in carbon dynamics. Earthworms had a significant positive effect on protease, invertase, urease and alkaline phosphatase activity, but were negatively correlated with β-cellobiohydrolase, β-glucosidase and xylosidase activity, suggesting that earthworms had different effects on different enzymes. Previous studies have shown that earthworm guts can secrete a large number of digestive enzymes (e.g., protease, amylase, lipase, cellulase and chitin) in casts to increase soil enzymatic activities (Pokarzhevskii, Van Straalen & Semenov, 2000; Shah, 2016). However, other studies have also suggested that the active enzymes in earthworm guts are inhibited, so the enzyme activity is low in the casts excreted by earthworms (McGill & Cole, 1981). Our results showed that earthworms had different effects on different enzymes, indicating that these enzymes were not directly excreted from earthworm guts and were instead derived from microorganisms in the soil. Generally, enzyme activity was significantly positively correlated with the quantity of microorganisms, and earthworms influenced enzyme activity by affecting microorganisms. In the absence of earthworms, microorganisms were the main driving force for cellulose breakdown (Nielsen et al., 2011). Under aerobic conditions, cellulose decomposition mainly depended on fungi and actinomycetes, and under anoxic conditions, cellulose was almost completely digested by bacteria. Aerobic cellulose decomposition resulted in more extracellular release than anaerobic cellulose decomposition. More extracellular enzymes are released under aerobic conditions than under anaerobic conditions (Trivedi, Anderson & Singh, 2013). Since the residues covered the soil surface, cellulose decomposed under aerobic conditions, so β-cellobiohydrolase, β-glucosidase and xylosidase activity was highest in the treatment with residues and without earthworms. This indicated that there was an increase in the fungal activity of this treatment’s soil and that these enzymes may be mainly produced by soil fungi. Some fungi such as trichoderma and pythium release various enzymes, including β-cellobiohydrolase, β-glucosidase and xylosidase (Hayano & Tubaki, 1985; Hayano, 1986). However, β-cellobiohydrolase, β-glucosidase and xylosidase activity decreased in the soil with earthworms, suggesting that these cellulases were not directly derived from earthworms and that the amount of soil fungi secreting these enzymes was reduced. In the absence of fungi, earthworms play a more important role in the cellulolytic decomposition of residues through their digestion process since cellulose is a part of their diet. Our results on days 28 and 126 also showed that protease, urease and alkaline phosphatase activity increased in the soil with earthworms. These enzyme activities were higher than in the soil without earthworms, perhaps due to an increased number of microorganisms secreting these enzymes into the soil with earthworms. Dash, Mishra & Behera (1979) observed earthworms eating fungi, actinomycetes, and bacteria, but when these microorganisms passed through the gut, they destroyed fungal spores, killing most of the fungi. This reduced the number of the actinomycetes and increased the amount of bacteria since they could adapt to the anaerobic environment of the gut. However, the increase in water-soluble carbohydrates and available nutrients in the casts discharged from the earthworms stimulated the rapid growth and reproduction of some microorganisms and bacteria. This proved the presence of microbial succession during the earthworms’ cellulose decomposition. Other studies have shown that chitin is the main component of the fungal cell wall, which earthworms may digest (Parle, 1963). However, our results did not show the regulatory effects of earthworms on chitinase activity.

## Conclusion

Our results confirmed that earthworm activity can significantly accelerate the degradation of rice residues and increase the content of soil macroaggregates that improve soil structure. We concluded that providing exogenous plant residues as food allows earthworms to convert plant carbon into soil carbon because they directly feed on rice residues and excrete casts that form soil aggregates to maintain SOC. During this process, the organic carbon content in the casts was higher than in the soil without earthworms, showing that earthworms have a main role in carbon stabilization. However, in the absence of exogenous plant residues, earthworms activated the original carbon in the soil and accelerated its degradation and consumption. The SOC content in the casts was lower than in the soil without earthworms, indicating that earthworms were key in promoting SOC mineralization. Earthworms directly feed on and digest rice residues, carrying out the primary decomposition of rice residues. The earthworms converted refractory residues into carbohydrates that were easily used by microorganisms, provided nutrients and accelerated the degradation of residues. When there were no earthworms in the soil, the degradation of residues was slower and depended mainly on microorganisms, particularly fungi. Moreover, earthworm activity transformed soil ecosystems with slower fungal-based nutrient turnover into systems that were bacterial-based with rapid nutrient turnover. We suggest that future studies focus on using earthworms to accelerate the degradation of rice or wheat residues returned to the farmland by: (1) cultivating more earthworms in the farmland; (2) monitoring the dynamics among soil aggregates, SOC, and earthworms after returning residues; (3) measuring CO_2_ emission and total carbon trends after returning residues to soil with earthworms; and (4) evaluating the effect of earthworm activity on soil microorganisms and pathogens after returning residues.

## Supplemental Information

10.7717/peerj.9870/supp-1 Supplemental Information 1 Supplemental Tables.

10.7717/peerj.9870/supp-2 Supplemental Information 2 Raw data.

## Additional Information and Declarations

### Data Availability

The following information was supplied regarding data availability: The data and code are available in the Supplemental Files.
