# Supplementary material for: Earthworms accelerate rice straw decomposition and maintenance of soil organic carbon dynamics in rice agroecosystems
Source: PeerJ. 2020 Sep 17;8:e9870. doi: 10.7717/peerj.9870 (PMC7502234; doi:10.7717/peerj.9870)
Supplement: Supplemental Information 1 [file peerj-08-9870-s001.docx]

**Supplemental file 1**

**Earthworms accelerate rice straw decomposition and maintenance of soil organic carbon dynamics in rice agroecosystems**

Ke Song^1^, Lijuan Sun^1^, Weiguang Lv^1^, Xianqing Zheng^1^, Yafei Sun^1^, William Terzaghi^2^, Qin Qin^1^, Yong Xue^1^

^1^Eco-Environmental Protection Research Institute, Shanghai Academy of Agricultural Sciences, Shanghai, China

^2^Department of Biology, Wilkes University, Wilkes-Barre, the United States of America

***Corresponding authors**

Yong Xue

Tel: +86 15921934521

Fax: +86 021 62202594

E-mail address: [exueyong211@163.com](mailto:exueyong211@163.com)

Qin Qin

Tel: +86 15021532267

Fax: +86 021 62202594

E-mail address: qinqin@saas.sh.cn

**Supplementary Table S1-1**. Soil organic carbon (SOC) in different treatments at Day 28 as affected by residues and earthworms. Treatments are: -Rr-Ew: without surface rice residues or earthworms, -Rr+Ew: without surface rice residues with earthworms, +Rr-Ew: with surface rice residues without earthworms, +Rr+Ew: with surface rice residues and with earthworms. R1-3 are 3 reduplicates in a treatment.

| Treatments | R1 | R2 | R3 |
| --- | --- | --- | --- |
| -Rr-Ew | 19.352 | 19.610 | 20.027 |
| -Rr+Ew | 17.301 | 17.402 | 18.588 |
| +Rr-Ew | 20.801 | 20.761 | 20.361 |
| +Rr+Ew | 22.105 | 21.863 | 23.191 |

**Supplementary Table S1-2**. Soil organic carbon (SOC) in different treatments at Day 126 as affected by residues and earthworms. Treatments are described in Supplementary Table S1-1. R1-3 are 3 reduplicates in a treatment.

| Treatments | R1 | R2 | R3 |
| --- | --- | --- | --- |
| -Rr-Ew | 19.367 | 19.032 | 19.497 |
| -Rr+Ew | 17.732 | 17.711 | 16.07 |
| +Rr-Ew | 20.717 | 21.521 | 21.764 |
| +Rr+Ew | 21.225 | 21.038 | 23.054 |

**Supplementary Table S2-1**. Aggregate content at Day 28 as affected by residues and earthworms. Treatments are described in Supplementary Table S1-1. R1-3 are 3 reduplicates in a treatment.

| Treatments | Reduplicates | 250-2000 | 53-250 | <53 |
| --- | --- | --- | --- | --- |
| -Rr-Ew | R1 | 7.75 | 37.54 | 48.56 |
|  | R2 | 13.52 | 35.83 | 64.82 |
|  | R3 | 14.14 | 26.61 | 51.23 |
| -Rr+Ew | R1 | 32.06 | 35.48 | 32.46 |
|  | R2 | 38.88 | 39.37 | 21.75 |
|  | R3 | 42.27 | 30.95 | 26.78 |
| +Rr-Ew | R1 | 11.96 | 42.31 | 37.33 |
|  | R2 | 10.24 | 42.47 | 38.4 |
|  | R3 | 20.86 | 51.35 | 39.86 |
| +Rr+Ew | R1 | 26.06 | 35.41 | 38.53 |
|  | R2 | 42.82 | 38.64 | 18.54 |
|  | R3 | 35.39 | 46.07 | 18.54 |

**Supplementary Table S2-2**. Aggregate content at Day 126 as affected by residues and earthworms. Treatments are described in Supplementary Table S1-1. R1-3 are 3 reduplicates in a treatment.

| Treatments | Reduplicates | 250-2000 | 53-250 | <53 |
| --- | --- | --- | --- | --- |
| -Rr-Ew | R1 | 10.27 | 23.56 | 66.17 |
|  | R2 | 13.29 | 34.72 | 51.99 |
|  | R3 | 19.42 | 42.74 | 37.84 |
| -Rr+Ew | R1 | 54.54 | 24.06 | 21.4 |
|  | R2 | 55.82 | 21.77 | 26.41 |
|  | R3 | 59.06 | 15.87 | 21.07 |
| +Rr-Ew | R1 | 27.11 | 35.68 | 33.21 |
|  | R2 | 31.74 | 31.36 | 38.9 |
|  | R3 | 27.81 | 40.19 | 34 |
| +Rr+Ew | R1 | 56.18 | 25.94 | 17.88 |
|  | R2 | 62.79 | 23.96 | 13.25 |
|  | R3 | 59.77 | 25.32 | 14.91 |

**Supplementary Table S3-1**. Aggregate-associated carbon (AC) at Day 28 as affected by residues and earthworms. Treatments are described in Supplementary Table S1-1. R1-3 are 3 reduplicates in a treatment.

| Treatments | Reduplicates | 250-2000 | 53-250 | <53 |
| --- | --- | --- | --- | --- |
| -Rr-Ew | R1 | 14.528 | 11.011 | 2.634 |
|  | R2 | 13.793 | 12.673 | 2.226 |
|  | R3 | 14.362 | 10.334 | 3.476 |
| -Rr+Ew | R1 | 12.533 | 10.743 | 2.863 |
|  | R2 | 12.013 | 10.073 | 2.116 |
|  | R3 | 12.337 | 9.867 | 1.156 |
| +Rr-Ew | R1 | 14.527 | 12.205 | 3.201 |
|  | R2 | 14.990 | 12.312 | 2.960 |
|  | R3 | 14.781 | 13.378 | 4.133 |
| +Rr+Ew | R1 | 16.002 | 12.629 | 1.853 |
|  | R2 | 15.956 | 12.830 | 1.902 |
|  | R3 | 16.482 | 10.659 | 2.636 |

**Supplementary Table S3-2**. Aggregate-associated carbon (AC) at Day 126 as affected by residues and earthworms. Treatments are described in Supplementary Table S1-1. R1-3 are 3 reduplicates in a treatment.

| Treatments | Reduplicates | 250-2000 | 53-250 | <53 |
| --- | --- | --- | --- | --- |
| -Rr-Ew | R1 | 13.882 | 10.113 | 2.336 |
|  | R2 | 13.720 | 10.520 | 1.974 |
|  | R3 | 13.983 | 10.361 | 1.989 |
| -Rr+Ew | R1 | 11.101 | 8.225 | 1.111 |
|  | R2 | 11.432 | 8.809 | 1.492 |
|  | R3 | 13.024 | 8.066 | 2.612 |
| +Rr-Ew | R1 | 15.932 | 13.704 | 3.012 |
|  | R2 | 15.775 | 13.209 | 3.782 |
|  | R3 | 15.976 | 14.386 | 2.735 |
| +Rr+Ew | R1 | 16.804 | 12.992 | 2.104 |
|  | R2 | 16.720 | 12.129 | 2.783 |
|  | R3 | 15.860 | 11.240 | 1.148 |

**Supplementary Table S4**. Soil basal respiration measured weekly as affected by residues and earthworms. Treatments are described in Supplementary Table S1-1. R1-3 are 3 reduplicates in a treatment.

| week | -Rr-Ew | | | -Rr+Ew | | | +Rr-Ew | | | +Rr+Ew | | |
| --- | --- | --- | --- | --- | --- | --- | --- | --- | --- | --- | --- | --- |
|  | R1 | R2 | R3 | R1 | R2 | R3 | R1 | R2 | R3 | R1 | R2 | R3 |
| 1 | 0.088 | 0.098 | 0.097 | 0.116 | 0.127 | 0.128 | 0.093 | 0.098 | 0.1 | 0.147 | 0.163 | 0.168 |
| 2 | 0.105 | 0.108 | 0.112 | 0.13 | 0.138 | 0.127 | 0.108 | 0.103 | 0.096 | 0.158 | 0.176 | 0.201 |
| 3 | 0.115 | 0.123 | 0.114 | 0.139 | 0.125 | 0.12 | 0.108 | 0.097 | 0.111 | 0.211 | 0.208 | 0.194 |
| 4 | 0.107 | 0.112 | 0.1 | 0.128 | 0.119 | 0.121 | 0.101 | 0.106 | 0.103 | 0.208 | 0.199 | 0.203 |
| 5 | 0.103 | 0.106 | 0.097 | 0.124 | 0.112 | 0.114 | 0.099 | 0.105 | 0.109 | 0.194 | 0.182 | 0.176 |
| 6 | 0.102 | 0.113 | 0.098 | 0.126 | 0.119 | 0.096 | 0.092 | 0.103 | 0.1 | 0.187 | 0.175 | 0.173 |
| 7 | 0.102 | 0.101 | 0.092 | 0.12 | 0.108 | 0.119 | 0.103 | 0.097 | 0.098 | 0.166 | 0.17 | 0.154 |
| 8 | 0.107 | 0.102 | 0.101 | 0.125 | 0.106 | 0.107 | 0.107 | 0.102 | 0.101 | 0.163 | 0.173 | 0.151 |
| 9 | 0.098 | 0.104 | 0.102 | 0.103 | 0.098 | 0.086 | 0.104 | 0.101 | 0.083 | 0.164 | 0.161 | 0.135 |
| 10 | 0.095 | 0.098 | 0.087 | 0.105 | 0.096 | 0.08 | 0.108 | 0.093 | 0.091 | 0.159 | 0.147 | 0.139 |
| 11 | 0.088 | 0.102 | 0.096 | 0.107 | 0.093 | 0.087 | 0.099 | 0.108 | 0.109 | 0.133 | 0.138 | 0.156 |
| 12 | 0.09 | 0.096 | 0.093 | 0.095 | 0.092 | 0.073 | 0.1 | 0.093 | 0.105 | 0.141 | 0.133 | 0.122 |
| 13 | 0.087 | 0.093 | 0.094 | 0.093 | 0.078 | 0.083 | 0.107 | 0.108 | 0.08 | 0.137 | 0.121 | 0.118 |
| 14 | 0.09 | 0.093 | 0.085 | 0.091 | 0.076 | 0.085 | 0.113 | 0.098 | 0.111 | 0.122 | 0.107 | 0.12 |
| 15 | 0.087 | 0.089 | 0.083 | 0.098 | 0.079 | 0.069 | 0.107 | 0.114 | 0.116 | 0.108 | 0.113 | 0.092 |
| 16 | 0.088 | 0.094 | 0.083 | 0.091 | 0.082 | 0.06 | 0.123 | 0.107 | 0.119 | 0.106 | 0.099 | 0.084 |
| 17 | 0.093 | 0.091 | 0.069 | 0.086 | 0.086 | 0.055 | 0.117 | 0.104 | 0.104 | 0.092 | 0.083 | 0.087 |
| 18 | 0.085 | 0.088 | 0.077 | 0.085 | 0.074 | 0.074 | 0.112 | 0.118 | 0.11 | 0.094 | 0.081 | 0.081 |

**Supplementary Table S5**. Dissolved organic carbon (DOC) measured weekly as affected by residues and earthworms. Treatments are described in Supplementary Table S1-1. R1-3 are 3 reduplicates in a treatment.

| week | -Rr-Ew | | | -Rr+Ew | | | +Rr-Ew | | | +Rr+Ew | | |
| --- | --- | --- | --- | --- | --- | --- | --- | --- | --- | --- | --- | --- |
|  | R1 | R2 | R3 | R1 | R2 | R3 | R1 | R2 | R3 | R1 | R2 | R3 |
| 1 | 162.153 | 165.457 | 164.331 | 171.352 | 175.227 | 173.857 | 162.312 | 165.897 | 164.753 | 179.583 | 178.122 | 174.67 |
| 2 | 164.328 | 160.345 | 161.442 | 180.364 | 183.221 | 183.347 | 168.336 | 165.342 | 162.458 | 191.352 | 186.005 | 185.054 |
| 3 | 164.556 | 162.573 | 164.191 | 176.568 | 177.774 | 181.519 | 164.238 | 164.871 | 157.232 | 188.468 | 193.36 | 189.64 |
| 4 | 160.224 | 163.57 | 160.871 | 175.265 | 176.783 | 171.015 | 164.01 | 165.913 | 161.793 | 185.471 | 186.368 | 178.272 |
| 5 | 160.236 | 162.362 | 154.394 | 174.322 | 176.448 | 165.744 | 164.053 | 163.83 | 171.13 | 180.352 | 182.015 | 172.977 |
| 6 | 158.721 | 156.255 | 155.628 | 174.286 | 169.11 | 161.637 | 165.992 | 167.921 | 158.702 | 178.39 | 172.674 | 172.406 |
| 7 | 155.092 | 158.367 | 159.705 | 171.352 | 167.883 | 167.835 | 163.67 | 165.882 | 159.653 | 175.21 | 174.562 | 181.976 |
| 8 | 148.368 | 156.437 | 163.293 | 165.445 | 169.228 | 167.363 | 160.573 | 162.041 | 155.555 | 174.11 | 167.452 | 167.149 |
| 9 | 155.09 | 153.772 | 158.909 | 158.356 | 160.221 | 171.29 | 160.365 | 164.682 | 158.775 | 167.349 | 163.853 | 173.8 |
| 10 | 154.226 | 157.824 | 158.907 | 158.225 | 159.338 | 166.272 | 165.884 | 157.901 | 160.213 | 161.427 | 165.338 | 166.526 |
| 11 | 154.112 | 153.57 | 151.563 | 162.485 | 158.442 | 148.238 | 160.337 | 162.885 | 152.174 | 158.132 | 159.356 | 153.868 |
| 12 | 150.226 | 154.378 | 152.241 | 157.34 | 158.93 | 146.118 | 160.334 | 162.653 | 152.919 | 158.227 | 160.352 | 152.869 |
| 13 | 152.011 | 154.683 | 149.397 | 150.36 | 147.03 | 147.642 | 158.901 | 164.078 | 158.153 | 158.42 | 153.557 | 156.672 |
| 14 | 152.116 | 153.672 | 148.025 | 145.113 | 148.224 | 145.417 | 159.044 | 162.388 | 151.432 | 150.16 | 158.336 | 160.627 |
| 15 | 153.063 | 151.844 | 147.714 | 143.778 | 148.369 | 143.172 | 160.115 | 157.446 | 157.131 | 153.275 | 153.683 | 155.691 |
| 16 | 147.864 | 150.236 | 146.222 | 144.582 | 147.88 | 146.659 | 156.772 | 158.04 | 154.551 | 152.11 | 156.372 | 154.617 |
| 17 | 146.348 | 151.347 | 149.285 | 146.237 | 142.116 | 141.35 | 156.362 | 153.833 | 155.816 | 150.683 | 154.778 | 140.129 |
| 18 | 147.303 | 145.678 | 147.795 | 142.035 | 144.458 | 139.868 | 155.773 | 156.168 | 156.876 | 146.793 | 148.002 | 149.431 |

**Supplementary Table S6-1**. Ergosterol content in different treatments at Day 28 as affected by residues and earthworms. Treatments are described in Supplementary Table S1-1. R1-3 are 3 reduplicates in a treatment.

| Ergosterol | R1 | R2 | R3 |
| --- | --- | --- | --- |
| -Rr-Ew | 34.58 | 42.33 | 32.45 |
| -Rr+Ew | 24.1 | 28.46 | 14.08 |
| +Rr-Ew | 136.45 | 115.99 | 133.97 |
| +Rr+Ew | 31.04 | 20.76 | 33.83 |

**Supplementary Table S6-2**. Ergosterol content in different treatments at Day 126 as affected by residues and earthworms. Treatments are described in Supplementary Table S1-1. R1-3 are 3 reduplicates in a treatment.

| Ergosterol | R1 | R2 | R3 |
| --- | --- | --- | --- |
| -Rr-Ew | 50.44 | 57.48 | 53.94 |
| -Rr+Ew | 17.55 | 12.27 | 10.8 |
| +Rr-Ew | 210.67 | 231.43 | 201.5 |
| +Rr+Ew | 13.47 | 18.76 | 13.38 |

**Supplementary Table S7**. The activities of protease, invertase, urease, alkaline phosphatase, β-cellobiohydrolase, β-glucosidase, xylosidase and chitinase enzymes in soil after 28 and 126 days of treatment as affected by the presence of residues and earthworms. Treatments are described in Supplementary Table S1-1. R1-3 are 3 reduplicates in a treatment.

| Enzyme | Days | Reduplicates | -Rr-Ew | -Rr+Ew | +Rr-Ew | +Rr+Ew |
| --- | --- | --- | --- | --- | --- | --- |
| Protease | 28 | R1 | 69.47 | 108.38 | 93.48 | 156.33 |
|  |  | R2 | 72.11 | 117.54 | 93.24 | 136.24 |
|  |  | R3 | 81.76 | 113.27 | 83.32 | 129.13 |
|  | 126 | R1 | 52.89 | 78.90 | 90.33 | 114.32 |
|  |  | R2 | 60.13 | 86.56 | 102.25 | 106.36 |
|  |  | R3 | 75.51 | 101.43 | 85.89 | 117.52 |
| Invertase | 28 | R1 | 940.18 | 1563.10 | 1772.11 | 2230.16 |
|  |  | R2 | 954.24 | 1480.34 | 1863.25 | 2405.78 |
|  |  | R3 | 1010.92 | 1159.24 | 1797.65 | 2390.91 |
|  | 126 | R1 | 748.22 | 1632.05 | 1993.25 | 2355.36 |
|  |  | R2 | 770.19 | 1598.38 | 2104.29 | 2498.35 |
|  |  | R3 | 774.51 | 1759.88 | 2163.75 | 2744.59 |
| Urease | 28 | R1 | 48.22 | 86.36 | 65.77 | 109.87 |
|  |  | R2 | 43.12 | 84.31 | 54.24 | 96.47 |
|  |  | R3 | 37.14 | 96.11 | 62.35 | 101.72 |
|  | 126 | R1 | 52.05 | 87.44 | 78.33 | 112.44 |
|  |  | R2 | 41.47 | 99.41 | 72.68 | 101.26 |
|  |  | R3 | 37.14 | 84.45 | 64.97 | 99.60 |
| Alkaline phosphatase | 28 | R1 | 74.33 | 157.88 | 135.63 | 177.88 |
|  |  | R2 | 76.29 | 174.24 | 142.09 | 182.58 |
|  |  | R3 | 85.40 | 157.97 | 112.44 | 205.50 |
|  | 126 | R1 | 73.32 | 160.23 | 151.74 | 209.93 |
|  |  | R2 | 85.45 | 148.38 | 158.29 | 188.75 |
|  |  | R3 | 82.35 | 162.07 | 148.56 | 179.49 |
| β-Cellobiohydrolase | 28 | R1 | 100.99 | 45.62 | 128.44 | 112.19 |
|  |  | R2 | 75.05 | 62.79 | 119.36 | 110.50 |
|  |  | R3 | 83.44 | 48.56 | 128.59 | 103.27 |
|  | 126 | R1 | 84.01 | 46.60 | 151.27 | 61.37 |
|  |  | R2 | 66.76 | 40.32 | 133.59 | 56.38 |
|  |  | R3 | 72.08 | 47.88 | 158.40 | 72.19 |
| β-Glucosidase | 28 | R1 | 144.33 | 93.69 | 199.39 | 149.30 |
|  |  | R2 | 138.91 | 78.58 | 230.45 | 180.33 |
|  |  | R3 | 153.80 | 92.11 | 204.11 | 189.36 |
|  | 126 | R1 | 115.30 | 86.78 | 289.19 | 133.45 |
|  |  | R2 | 119.36 | 82.18 | 244.76 | 142.39 |
|  |  | R3 | 132.46 | 83.14 | 238.59 | 125.33 |
| Xylosidase | 28 | R1 | 77.41 | 32.26 | 115.89 | 84.12 |
|  |  | R2 | 67.59 | 42.12 | 102.58 | 89.16 |
|  |  | R3 | 71.34 | 56.49 | 95.34 | 83.47 |
|  | 126 | R1 | 48.80 | 28.26 | 158.48 | 58.77 |
|  |  | R2 | 62.66 | 35.79 | 142.79 | 58.62 |
|  |  | R3 | 64.38 | 42.34 | 163.80 | 73.48 |
| Chitinase | 28 | R1 | 122.56 | 119.81 | 127.99 | 109.32 |
|  |  | R2 | 118.36 | 126.84 | 123.07 | 121.46 |
|  |  | R3 | 130.22 | 117.44 | 140.48 | 126.89 |
|  | 126 | R1 | 126.21 | 138.73 | 140.33 | 115.41 |
|  |  | R2 | 120.49 | 104.76 | 120.36 | 134.11 |
|  |  | R3 | 105.48 | 115.32 | 117.47 | 123.57 |

**Supplementary Table S8**. The results of a two-way ANOVA comparing the four treatments regarding SOC, Aggregates, Aggregate carbon and Ergosterol content. Treatments are described in Supplementary Table S1-1. Rr means Rice residues, Ew means Earthworms. F values are shown in the table. Treatments indicated by the same letter are not significantly different at P ≤ 0.05 on the basis of a two-way ANOVA.

| Items | Traits | Days | Rr | Ew | Rr*Ew |
| --- | --- | --- | --- | --- | --- |
| SOC | \ | 28 | 53.46* | 1.81 | 17.67* |
|  |  | 126 | 42.16* | 1.17 | 1.92 |
| Aggregates | >250 mm | 28 | 0.004 | 45.21* | 0.64 |
|  |  | 126 | 21.12* | 358.95* | 8.88* |
|  | 53-250 mm | 28 | 7.79* | 0.32 | 1.46 |
|  |  | 126 | 0.99 | 12.93* | 0.14 |
|  | <53 | 28 | 4.12 | 21.26* | 2.65 |
|  |  | 126 | 7.85* | 32.15* | 1.09 |
| Aggregate carbon | >250 mm | 28 | 162.37* | 2.57 | 92.50* |
|  |  | 126 | 97.40* | 4.59 | 14.65* |
|  | 53-250 mm | 28 | 8.22* | 2.48 | 0.23 |
|  |  | 126 | 118.18* | 29.81* | 0.23 |
|  | <53 | 28 | 0.95 | 7.24* | 0.56 |
|  |  | 126 | 3.37 | 4.31 | 1.2 |
| Ergosterol content | \ | 28 | 115.26* | 155.18* | 87.58* |
|  |  | 126 | 292.88* | 639.52* | 280.99* |

**Supplementary Table S9**. The results of a two-way ANOVA comparing the four treatments regarding the enzymes activities. Treatments are described in Supplementary Table S1-1. Rr means Rice residues, Ew means Earthworms. F values are shown in the table. Treatments indicated by the same letter are not significantly different at P ≤ 0.05 on the basis of a two-way ANOVA.

| Enzyme | Days | Rr | Ew | Rr*Ew |
| --- | --- | --- | --- | --- |
| Protease | 28 | 18.83* | 80.73* | 1.45 |
|  | 126 | 23.47* | 17.21* | 0.31 |
| Invertase | 28 | 162.91* | 47.54* | 0.5 |
|  | 126 | 268.4* | 101.01* | 11.49* |
| Urease | 28 | 19.97* | 153.69* | 0.35 |
|  | 126 | 25.1* | 87.69* | 2.91 |
| Alkaline phosphatase | 28 | 30.14* | 105.27* | 3.49 |
|  | 126 | 96.96* | 111.93* | 11.11* |
| β-cellobiohydrolase | 28 | 87.91* | 25.16* | 2.92 |
|  | 126 | 78.39* | 120.3* | 28.2* |
| β-glucosidase | 28 | 80.19* | 32.54* | 1.31 |
|  | 126 | 111.25* | 85.6* | 23.78* |
| Xylosidase | 28 | 57.07* | 23.24* | 0.92 |
|  | 126 | 147.88* | 125.05* | 44.39* |
| Chitinase | 28 | 0.29 | 2.52 | 1.08 |
|  | 126 | 0.82 | 0 | 0.07 |

**Supplementary Table S10**. Soil organic carbon (SOC) in different treatments at three time-points as affected by residues and earthworms. Values are means, n = 3. Treatments indicated by the same letter are not significantly different at P ≤ 0.05 on the basis of one-way ANOVA.

| Treatments | Day 0 | Day 28 | Day 126 |
| --- | --- | --- | --- |
| -Rr-Ew | 19.877 a | 19.663 a | 19.299 a |
| -Rr+Ew | 19.877 a | 17.764 b | 17.171 b |
| +Rr-Ew | 19.877 b | 20.641 ab | 21.334 a |
| +Rr+Ew | 19.877 b | 22.386 a | 21.772 a |
